# Unsupervised type III polygraphy in children undergoing adenotonsillectomy: a technical and economic report

**DOI:** 10.5935/1984-0063.20200094

**Published:** 2021

**Authors:** Iury Lima Veloso, Camila de Castro Corrêa, José Vicente Tagliarini, Silke Anna Theresa Weber

**Affiliations:** 1 Botucatu Medical School - State University São Paulo, UNESP, Department of Ophthalmology and Otorhinolaryngology - Botucatu - Sao Paulo - Brazil.; 2 University of Brasília, UnB - Brasília - Distrito Federal - Brazil.; 3 Plateau University Center of Distrito Federal, UNIPLAN - Brasília - Distrito Federal - Brazil.

**Keywords:** Adenoids, Palatine Tonsil, Polysomnography, Sleep Apnea, Obstructive, Sleep Medicine Specialty

## Abstract

**Objective:**

To evaluate the economic and technical viability of the sleep study (type III) in children with adenotonsilar hypertrophy.

**Methods:**

141 children were submitted to sleep study (type III), aged between three and 11, all with symptoms of OSA. The frequency of failed examinations and a comparison of cost analysis between complete polysomnography were described.

**Results:**

41 exams lost at least one sensor. The sensor with the highest number of losses was the oximetry, observed in 14.28%. The 100 valid sleep studies allowed the diagnosis of severe OSA in 36 children. Sleep study accounts for approximately 63% of the value of the PSG type I, thus, it showed to be cost effective even with the repetition of the failed one.

**Conclusion:**

Sleep study (type III) may have high failure rates and it was a reliable exam for the identification of severe OSA. The cost analysis showed economic feasibility, even with a high failure rate and necessity of repetition.

## INTRODUCTION

Sleep disordered breathing and obstructive sleep apnea (OSA) have a high impact on morbidity among children and can lead to learning, behavioral, and developmental disturbances^1, 2^. It is mainly related to the increase of the size of the tonsils, and surgical removal (adenotonsillectomy) is the treatment of choice for children^3, 4^, although craniofacial disorders and obesity are important risk factors for pediatric OSA. The complete polysomnography (PSG) with technical supervision (type I) is the gold standard for OSA diagnosis, while the out of center sleep test is usually reserved for children and adults without any comorbidities and with a high clinical suspicion of OSA. As an alternative sleep study, actually, there are considered three levels as valid, all being performed without the supervision of a technician, being the type II a sleep study which monitors minimally EEG, ECG, EOG, respiratory flow and movement, oxygen saturation, and body position. The type III sleep study should monitor at least four parameters, including respiratory flow and movement, heart rate, oxygen saturation, actigraphy, snoring, besides others. The type IV sleep study monitors two to four parameters, as oximetry, respiratory flow, heart rate, besides others, and is not recommended^4, 5, 6^.

With the increased interest in sleep disorders and growing demand for their investigation and treatment, the feasibility of PSG type I, complete supervised in-laboratory exam, for all suspected patients has proven to be impracticable, due to high cost or lack of availability of pediatric sleep labs^7^. The type III sleep study is suggested as an alternative, and in recent studies, it has shown good results as compared to PSG type I, even without the scoring impact of events related to arousals^8, 9^.

Actually, there is a constant increase in the waiting list to PSG type I in pediatric habilitated laboratories, causing a delay in the diagnostic and resulting in more adverse effects^10, 11^. In children with a recognized high risk of OSA, either by physical examination or radiological findings, after the identification of possible comorbidities and risk factors to the persistence of OSA, the objective assessment of the severity of the disease before surgical intervention is advocated. When PSG type I is unavailable, an alternative sleep study is recommended^3^. The type III sleep study has been proposed as the quickest and most accessible way to investigate sleep apnea, with an upside of possible cost reduction^8, 9^, but despite the increasing use of a type III sleep study, there is still insufficient data to consolidate the recommendation of the type III sleep study to the detriment of the study in the laboratory^10^.

In this study, we hypothesized that the type III sleep study is technically viable and can be used as a diagnostic tool in OSA children. Thus, the objective of the study was to evaluate the technical (failure frequency) and economic feasibility of the type III sleep study in children with snoring and adenotonsillar hypertrophy to help decision making of the best treatment and perioperative planning.

## MATERIAL AND METHODS

The study was approved in the local research ethics committee with the number CAAE: 42976815.0.0000.5411, all parents/guardians signed informed written consent.

Children aged between three and 11 with clinical signs and symptoms of OSA and indication of adenotonsillectomy were invited to participate in this study. All children complaint of snoring, restless sleep, mouth breathing, and otolaryngologic examination showed tonsils size 3 or 4 (Brodsky scale) and /or adenoids occupying 70% or more of the rhinopharynx. Patients with neurological diseases, other comorbidities and/or genetic syndromes were excluded. The performance of a type III sleep study was proposed on the time of surgery at the surgical ward the night before. As our sleep unit had one porTable equipment, only one exam was performed per night, even having two children planned for surgery on most of the nights. Children were invited by convenience during the period of January 2014 to December 2016, monitoring about 50% of the children undergoing adenotonsillectomy during this period. Then non-monitored children were similar in the otolaryngologic exam as tonsil size and clinical complaints, with no parental refusal. 141 children underwent a type III sleep study, using the Philips StarDust II® equipment. The equipment was placed by a trained otolaryngology resident or physical therapist in the late afternoon, at the end of a normal day shift.

The assembly and scoring were performed according to the standards of the pediatric criteria of the American Academy of Sleep Medicine (AASM) by a medical specialist in sleep medicine of the Sleep Respiratory Disorders service^12^. The sensor data were considered valid when showing at least four hours of recording. The following data were obtained from the exam: sensor record time, apnea-hypopnea index (AHI), obstructive apnea index-OAI, mean and minimum saturation, to characterize the severity of obstructive respiratory disorders. An AHI >1 event per hour was considered positive for OSA, an AHI >10e/h as severe OSA^13^.

As the first outcome, the technical feasibility of the type III sleep study was analyzed. We considered failure, the exam with less than four hours of recording, loss of channels with less than four hours recording time, and oxygen saturation signal with less than four hours duration. The frequency of failed examinations, failures per sensor, and loss of performance were described. As the second outcome, a cost analysis of complete PSG and the type III sleep study was carried out, comparing the direct costs of consumables.

### Statistical analysis

The population size was determined by convenience, no previous sample size calculation was performed. Statistical analysis considered the significance of *p*<0.05. The results were presented as the mean ± standard deviation (SD).

The data were tabulated for the frequency of loss for each sensor and the comparison between failures of each sensor was performed by using Fisher’s exact test. The comparison by age group and failure of the sensors was performed by chi-squared test.

The relative risk of younger aged children (preschool) to lose a sensor was estimated using the formula (A/(A+B))/ (C/(C+D)), considering A as the number of preschool-aged children who lost a sensor, B as preschool ones with no loss, C school-aged children who lost a sensor, and D school-aged ones with no loss.

The analyses were made using SAS for Windows®, version 9.12.

## RESULTS

A total of 141 children, 76 males, mean age 6.38±2.46 were included. The distribution of the study population for demographic data (age and sex) is exposed in Graphic 1. The general failure rate in the exams was 29.08% (n=41), with no difference between sex (χ^2^=0.06; *p*=0.80). The sleep study showed the distribution of OSA severity in 3% as normal, 15% as mild, 26% as moderate, and in 56% as severe.

**Graphic 1:**
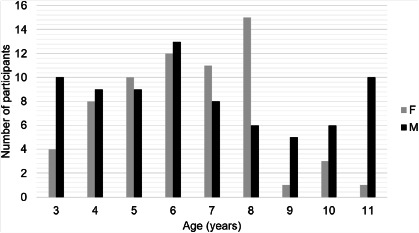
The distribution of the study population, considering the demographic characteristics age and sex.

The sensor with the highest number of losses was the one of the oximetry, observed in 14.28%, but with no significant difference when compared to the loss of the nasal cannula. The loss of more than one sensor was significantly lower than that of one isolated sensor (*p*=0.011). The thoracoabdominal belt, monitoring respiratory movement, showed valid results in all exams (Table 1).

**Table 1:** Frequency of failure for each sensor.

Lost sensor	N	% of the total of all exams	% of failed exams
None	100	70.92	
Oximeter	21	14.28	50
Nasal cannula	15	10.20	35.72
Both (oxymetry and nasal cannula)	5	3.55	12.20

Notes: n = Number of exams; % = Percentage.

Considering that the possibility of the failure of a sensor might be age- dependent, we separated the individuals according to two age groups (pre-school children: three to six years, elementary school children: seven to 11 years). The relative risk of failure in preschool children was almost 1.5 folds higher (Table 2).

**Table 2:** Distribution of the analyzed tests (with and without failure) by age group.

	Tests without fail		Tests failed	
	n	(%)	n	(%)
Pre-scholar	50	(50)	26	(63)
Scholar	50	(50)	15	(37)
Total	100	(100)	41	(100)

Notes: RR = Relative risk (A/(A+B))/(C/(C+D)).

The results of the type III sleep study showed a broad distribution of sleep disordered breathing, 36 children were diagnosed with severe OSA, with the planning of perioperative monitoring. The mean results of the valid type III sleep studies are exposed in Table 3.

**Table 3:** Results of the valid polysomnography.

	Mean±std dev	Median	Minimum	Maximum
ODI	16.7±14.9	11.9	0.1	76.4
SpO_2_ min	74.8±12.5	77	27	93

Notes: AHI = Apnea-hypopnea index; ODI = Obstructive desaturation index; SpO_2_ min = minimum saturation.

We listed the estimated costs of PSG type I and the type III sleep study, valid for a public health service in Brazil, taking into account the amounts paid by the Brazilian health system, *Sistema* Único *de Saúde (SUS).* Due to the long distances of family homes to the hospital, all exams were realized in the hospital, thus, for PSG type I and for the type III sleep study, the costs for hospitalization, procedure, and bedding were the same, in accordance with the SUS.

The costs of the consumable materials according to the values obtained in the last bidding of the institution were noted. The materials that are reused in several exams, as the oximeter and the thoracoabdominal belt for type III sleep studies, or electrodes, oximeter and respiratory belts for the PSG type I, had their value divided by the number of possible exams (Table 4). The costs of sterilization of reused equipment were not taken into account.

**Table 4:** Estimated cost involved in polysomnography type I and the type III sleep study.

	Unit price (R$)	Exam expense	Cost of the exam (R$)
**Type I polysomnography**			
Nasal cannula	13.00	1	13.00
Technician	395.00	÷ 3	131.66
Electrodes for ECG	0.60	× 4	2.40
Jelly for EEG	250.00	÷ 96	2.60
Hospitalization (SUS)	100.00	-	100.00
Procedure (SUS)	170.00	-	170.00
Bedding	21.00	-	21.00
Nasal thermistor	645.00	÷ 72	8.95
Electrodes	1500.00	÷ 72	20.80
Capnography	7000.00	÷ 144	48.6
Total:			519.01
**Type III polysomnography**			
Nasal cannula	13.00	1	13.00
Battery 9V	22.00	1	22.00
hospitalization (SUS)	100.00	-	100.00
Procedure (SUS)	170.00	-	170.00
Bedding	21.00	-	21.00
Total			326.00

Notes: SUS = Unique Health System of Brazil.

## DISCUSSION

In this study, we evaluated the risk of the failure of an unsupervised type III sleep study in children, focusing on the frequency and type of sensor lost, besides the economic analysis of the feasibility of realizing repetitive exams. In our study, the failure rate was high, suggesting the necessity of trained persons for installing the device. Despite the high failure rate, the repetition of the failed exam was still economically more feasible than the PSG type I.

The type III sleep study is performed by a portable device, without any direct intervention. For it to be valid, the sleep monitoring of at least six hours is necessary^13^. The type III sleep study is a low-cost, easy-to-perform exam, allowing for a larger number of exams. It is considered a reliable test for the diagnosis of OSA in adults, showing a strong correlation in any value of AHI^14^. However, there is still a lack of consensus of studies enrolling the pediatric and adult population^13, 15, 16, 17, 18, 19^.

In children, the most frequent sleep disorder is related to obstructive breathing, as primary snoring or OSA. Children, especially those with OSA, have a more restless sleep. Also, they show a lower collaboration to fix all sensors and electrodes than adults. Thus, the pediatric population certainly benefits from the easier-to-perform exam, besides increasing the facilities for realization in a more comforTable environment, at home.

Our study group showed a predominance of the preschool age group, both sexes, similar to the reported population submitted to adenotonsillectomy due to respiratory disorders in several studies of pediatric OSA. This strengthens the representativity of our study group for the real-world necessity of PSG prior to surgical OSA treatment^1, 4^.

The failure rate in the present study was 29.08%. Studies report failures varying between three and 33%^13, 16, 18^, however, some of these studies include equipment failure and not only loss of sensors during the night. There is still no consensus on this value, but we observed an improvement of failure rate in the second year of the study when our team was best trained, enhancing the importance of placing of the equipment by a trained person. Brockmann et al. (2013)^16^ also reported that the home performance of the type III sleep study in children, when assembled by a trained professional, gives similar results to the procedure performed in a hospital.

Initially, we expected a higher rate of non-valid tests in the younger children aged three to six years old, because they are more restless and more resistant to the installation and fixing of the electrodes and sensors, as reported by Scalzitti et al. (2017)^18^. However, our small number of enrolled children did not allow reaching statistical significance when failure rate was compared for different age groups^18^.

In our study, we evaluated the frequency of loss per sensor, considering the sensor of hemoglobin saturation (oximeter), the thoracoabdominal belt measuring the respiratory movement, and the nasal cannula of respiratory airflow. A slight predominance of oximeter loss was found in absolute numbers, but it showed not to be significant when compared to the nasal cannula. The lack of significance may be related to the small sample number. The nasal flow showed the second-highest failure rate. As reported by other authors, airflow detected by nasal thermistor cannula has already some limitations of detecting hypopnea, and certainly was worsened by the mouth breathing pattern of the child^8, 16, 17, 18^. When analyzing the simultaneous loss of nasal flow and oximetry during the exam, it was significantly lower than the isolated signal loss. Both sensors are more difficult to be positioned and fixed in children, thus, are more prone to be displaced during body movements. The investment in wireless sensors might help in the future.

Interestingly, the monitoring of respiratory movement by the thoracoabdominal belt was not lost on any examination. The child accepts the placing of the belt more easily and, as it is fixed to the body, it was not displaced during the night. In the literature, there is no other study that analyzes sensordifferentiated loss, but we consider this an important point in the evaluation to allow the guidance of any type of intervention to improve the exam’s success rate. The easy loss of any sensor at night and the exam not being supervised has been highlighted as the major limitation of the performance and validation of a type III sleep study^16, 17, 18^.

There are few studies comparing the financial aspects and the cost-effectiveness of portable sleep study^15, 17^, and it is argued that this aspect should be included in future studies. We evaluated the simplified estimated costs based on the payment schedules of the Brazilian public health system SUS for both types of exams.

As seen in Table 4, the sleep study accounts for approximately 63% of the value of the supervised exam. Based on these values, even with a failure rate as high as 30% and repeating the failed exam, the sleep study was still money saving in our study, what is important especially in low- and middle-income countries. Bruyneel and Ninane (2014)^20^ reported an estimated cost saving ranging from 25 to 50% for unsupervised sleep studies. Other authors also report cost saving, but they do not take into account a myriad of factors that interfere with costs, such as patient displacement, maintenance of equipment, cost related to technical personnel, or the number of exams that must be repeated due to failure^16, 17, 21^. The small data in the literature and the different methodology used for the economic evaluation of sleep studies turns an analysis of comparison difficult.

At our institution, we use the unsupervised type III sleep study for the diagnosis of OSA in children with tonsil hypertrophy and obstructive respiratory disorders in sleep, to assess the risk of postoperative complications in patients with severe OSA^3, 4, 9, 11, 16^. Due to mobility problems of the family, all exams were performed the night before surgery, which might have influenced results for more restless sleep and a shorter sleep duration, due to anxiety before the surgery. Nevertheless, in our series, a high rate of severe OSA was identified. This large number is certainly related to the characteristics of our service, receiving patients from a large area with long queues of referral and preference being given to clinically more severe patients. Although the literature suggests that the unsupervised sleep study underestimates the actual values of the AHI^9, 11^, in our service the examination achieved its purpose of identifying severe OSA children. These children were kept monitored at the hospital after surgery, reducing the risk of respiratory complications.

Our study has several limitations. Certainly, the small number of children enrolled hinders the statistical significance of some results. Almost half of the children submitted to surgery during the study period were included in this study, mostly due to technical problems as we disposed only of one equipment at that time, which might have enhanced the inclusion of children with more severe respiratory complaints. The study did not report the clinical characteristics of the children. Although we know that mostly young children and obese ones are at higher risk for complications, this research focused on the technical and economic feasibility of unsupervised type III sleep studies.

Beside all these limitations, we consider the type III sleep study as a feasible and cost-saving alternative for severe pediatric OSA diagnosis, enabling more children to be accessed, mostly in underserved environments.

## CONCLUSION

The unsupervised type III sleep study showed high failure rates for oximeter and nasal cannula. The placement of the equipment must be carried out by a trained professional, obeying a validated protocol. The cost analysis showed economic feasibility, even with a high failure rate and the necessity of a second exam.
